# Effects of Aircraft Noise on Sleep: Federal Aviation Administration National Sleep Study Protocol

**DOI:** 10.3390/ijerph20217024

**Published:** 2023-11-06

**Authors:** Mathias Basner, Ian Barnett, Michele Carlin, Grace H. Choi, Joseph J. Czech, Adrian J. Ecker, Yoni Gilad, Thomas Godwin, Eric Jodts, Christopher W. Jones, Marc Kaizi-Lutu, Jennifer Kali, Jean D. Opsomer, Sierra Park-Chavar, Michael G. Smith, Victoria Schneller, Nianfu Song, Pamela A. Shaw

**Affiliations:** 1 Unit for Experimental Psychiatry, Division of Sleep and Chronobiology, Department of Psychiatry, University of Pennsylvania Perelman School of Medicine, Philadelphia, PA 19104, USA; 2 Department of Biostatistics, Epidemiology, and Informatics, University of Pennsylvania Perelman School of Medicine, Philadelphia, PA 19104, USA; 3 Harris Miller Miller & Hanson Inc. (HMMH), Anaheim, CA 92805, USA; 4 Westat, Rockville, MD 20850, USA; 5 Center for Clinical Epidemiology & Biostatistics, University of Pennsylvania Perelman School of Medicine, Philadelphia, PA 19104, USA

**Keywords:** noise, sleep, awakening, aircraft, exposure–response, electrocardiogram, body movement, sleep disturbance

## Abstract

Aircraft noise can disrupt sleep and impair recuperation. The last U.S. investigation into the effects of aircraft noise on sleep dates back more than 20 years. Since then, traffic patterns and the noise levels produced by single aircraft have changed substantially. It is therefore important to acquire current data on sleep disturbance relative to varying degrees of aircraft noise exposure in the U.S. that can be used to check and potentially update the existing noise policy. This manuscript describes the design, procedures, and analytical approaches of the FAA’s National Sleep Study. Seventy-seven U.S. airports with relevant nighttime air traffic from 39 states are included in the sampling frame. Based on simulation-based power calculations, the field study aims to recruit 400 participants from four noise strata and record an electrocardiogram (ECG), body movement, and sound pressure levels in the bedroom for five consecutive nights. The primary outcome of the study is an exposure–response function between the instantaneous, maximum A-weighted sound pressure levels (dBA) of individual aircraft measured in the bedroom and awakening probability inferred from changes in heart rate and body movement. Self-reported sleep disturbance due to aircraft noise is the secondary outcome that will be associated with long-term average noise exposure metrics such as the Day–Night Average Sound Level (DNL) and the Nighttime Equivalent Sound Level (*L*_night_). The effect of aircraft noise on several other physiological and self-report outcomes will also be investigated. This study will provide key insights into the effects of aircraft noise on objectively and subjectively assessed sleep disturbance.

## 1. Introduction

### 1.1. Effects of Nocturnal Noise Exposure on Sleep and Health

Undisturbed sleep of sufficient length is of paramount importance for the maintenance of health and well-being [1]. The human auditory system has a watchman function and is constantly monitoring our environment for threats. Noise can be a potent disruptor of sleep and is considered one of the most detrimental environmental effects of air traffic [2].

According to a 2011 estimate by the World Health Organization (WHO), approximately 900,000 healthy life years are lost annually in the European Union due to sleep disturbance induced by environmental noise [3]. Epidemiologic studies associate long-term exposure to noise levels and cardiovascular disease risk (e.g., high blood pressure, heart attacks, stroke) [4,5]. Epidemiologic research also suggests that nocturnal noise exposure is more closely related to long-term health outcomes than daytime noise exposure, which may be due to the fact that people are typically at home during the night, while their daytime location may vary (e.g., at work) [6].

With regard to noise-related cardiovascular pathogenesis, two studies provide evidence for impaired flow-mediated dilation of the brachial artery after a single night of exposure to rail [7] or aircraft noise [8], suggesting that a single night of noise exposure can affect vascular function. The latter finding was replicated in a patient population with, or at high risk of, coronary artery disease [9]. Analyses of blood proteins from individuals exposed to rail noise [7] found noise-induced changes indicative of a pro-thrombotic and pro-inflammatory phenotype, and thus provide a molecular basis and biologic plausibility not only for the increased cardiovascular disease risks, as demonstrated by epidemiological studies, but also for other disease endpoints such as diabetes [10], neurodegenerative disease [11], obesity [10], and breast [12] and colon [13,14] cancer.

In one rodent study, negative effects on blood vessels and composition were primarily observed if the noise exposure was intermittent (like aircraft noise) and during the sleep phase [15], highlighting the relevance of intermittent nighttime noise exposure for health. A recent retrospective case-crossover study at Zurich airport demonstrated that aircraft noise exposure levels in the two hours preceding a cardiovascular event were associated with mortality [16]. Thus, nocturnal noise exposure may not only contribute to pathophysiological changes that increase cardiovascular disease risk but may also evoke physiological arousals that trigger a fatal event. Collectively, these studies corroborate the importance of an undisturbed sleep of sufficient duration for health and well-being.

### 1.2. Field Studies on the Effects of Aircraft Noise on Sleep

Several field studies have examined the effects of aircraft noise on sleep and have shown that the probability of awakening increases with the maximum A-weighted sound pressure level (*L*_AS,max_) of the aircraft event [17,18]. However, these studies have primarily been conducted in Europe, and due to differences in culture and housing structure, as well as operational procedures, the results from studies performed outside the United States (U.S.) may not translate directly to U.S. airports. Therefore, it is important that field studies be conducted in the U.S. to acquire current data on sleep disturbance relative to varying degrees of noise exposure.

With the most recent U.S. sleep study dating back to 1996 [19], U.S. research on the effects of aircraft noise on sleep has lagged over the past 25 years. During the intervening time, U.S. air traffic has changed, with changes in the number of operations on one hand, and significant reductions in noise levels of single aircraft on the other. In addition, most previous U.S. investigations into the effects of aircraft noise have relied on self-reported annoyance data obtained via surveys. Self-reported annoyance by aircraft noise is at best a proxy for objective sleep disturbance because annoyance is not exclusive to effects on sleep and is influenced by individual characteristics such as noise sensitivity and attitude to the noise source. Furthermore, sleep is, by definition, spent in an unconscious state, which makes it difficult to self-assess since the individual is unaware of how they are reacting to the noise unless they are fully awoken by the noise and recall this awakening the following morning. Since self-assessment leads to possible errors, objective measures are needed.

Furthermore, past U.S. studies on the effects of aircraft noise on sleep predominantly used the so-called “push button” methodology, where study participants were required to push a button whenever they woke during the night. This method has been shown to have low sensitivity, as most awakenings are too short for subjects to regain waking consciousness and initiate a response [20]. Therefore, most awakenings relevant for sleep recuperation are missed by this methodology, and physiologic measurements of sleep are needed. For this purpose, a new study methodology was developed and validated to measure noise-induced awakenings unobtrusively with a small device attached to the chest using only two electrodes (see Section 2.4.2) [21,22]. The device measures body movement and heart rate, two variables strongly associated with awakenings from sleep [21]. This methodology was piloted in two studies, one at Philadelphia International Airport (PHL) airport and one at Hartsfield–Jackson Atlanta International Airport (ATL) and was found to be feasible for conducting a larger-scale national study [23,24].

Apart from the two above-mentioned pilot studies, no previous study in the U.S. has collected physiologic data (e.g., body movement or heart rate) and measured indoor aircraft noise levels in residents living in the proximity of airports. As such, the Federal Aviation Administration (FAA) National Sleep Study (NSS) discussed here is a unique and comprehensive study, and the first to go beyond a single or small number of selected airports. Since airports differ in nocturnal flight operations and patterns, it is necessary to investigate several airports across the U.S. that are representative of all U.S. airports with relevant nocturnal air traffic. Furthermore, the NSS collects information on measured, rather than estimated, levels of aircraft noise inside of residents’ bedrooms.

### 1.3. Regulatory Relevance

Current, objective, and high-fidelity information on the effects of aircraft noise on sleep is critical for assessing community impacts. The existing scientific data are outdated and lack the fidelity provided by modern data collection techniques. Failure to update data and relationships will cause the FAA to continue to rely on data that are at least 25 years old and continue to have the public and members of Congress question the validity of the current level of regulatory protection against adverse effects of aircraft noise on sleep.

Currently, the FAA defines a significant noise threshold as a Day–Night Average Sound Level (DNL) of 65 dB. In 1979, through the Aviation Safety and Noise Abatement Act (ASNA), the FAA implemented the ASNA’s provisions in 14 CFR Part 150. This regulation adopted the DNL metric and the 65 dB land use compatibility guideline. A DNL of 65 dB was chosen because it balanced environmental goals with technical and economic feasibility. In 1992, the Federal Interagency Committee On Noise (FICON) reevaluated the DNL and the 65 dB threshold and confirmed its continued use for the stated purpose [25]; this was the last in-depth government agency review on the metric and measure. In addition, even though the areas of DNL 65 dB contours around airports have decreased over the last 30 years, community opposition and challenges regarding aircraft noise have increased around many airports. This is corroborated by findings from the FAA’s Neighborhood Environmental Survey (NES), a representative survey of people living in the vicinity of 20 U.S. commercial airports, that was published in early 2021 and found significantly higher aircraft noise annoyance at the same DNL compared to earlier US surveys [26]. The FAA believes this is due in part to changes in aircraft operations. Although most of the commercial airline fleet is newer and quieter, the sheer number of flight operations (particularly at the largest and busiest airports) has increased over the last 30 years.

### 1.4. Objectives

The NSS collects nationally representative information on the effects of aircraft noise on sleep to derive exposure–response relationships between the *L*_AS,max_ of single aircraft noise events (ANEs), expressed in decibels (dB), and the likelihood of waking up, expressed as a percent chance (0–100%). Other measures of sleep disturbance and related health effects will also be analyzed as secondary endpoints.

A postal/web survey is used to gather data on eligibility and to recruit participants for the 5-night in-home field study. For survey respondents interested in and eligible for participation in the field study, sound recording and electrocardiography (ECG)/actigraphy equipment is shipped to them for objective measurements of noise-induced awakenings. At the end of the 5-night measurement period, equipment and surveys are shipped back by study participants for analysis.

## 2. Methodology and Materials

### 2.1. Study Design

#### 2.1.1. Overview

Power calculations based on 2018 traffic data (see Section 2.2) indicated that 77 U.S. airports met the criteria for relevant nighttime air traffic (see Section 2.1.3 for the definition of “relevant”) and were thus included in the sampling frame. Recruitment surveys (see Section 2.3.2 and Appendix A) are sent to randomly sampled addresses of stratified areas around the identified 77 airports. Sampling strata are based on predicted outdoor energy–average nighttime aircraft noise levels (*L*_night_) using 2018 traffic data (see Section 2.3.1).

Individuals who complete the Recruitment Survey, indicate their interest in participating in the study, and meet the eligibility criteria (see Section 2.3.3), are enrolled in the 5-night in-home field study (see Section 2.4) to collect noise and physiological (ECG and body movement) data, as well as complete questionnaires related to subject characteristics and daily morning sleep logs. Interested and eligible participants are consented into the study and then scheduled for a specific measurement week. A study manual (including additional surveys, detailed instructions, and step-by-step procedures; see Section 2.4.1) and study equipment (see Section 2.4.2) are mailed to participants in the week prior to the measurement week. Upon completion of the five nights, the manual and equipment are returned to the study team by mail. Awakenings from sleep will be inferred from ECG and movement data using a previously validated algorithm [22], and analyzed relative to measured noise levels in the bedroom (see Section 2.4.1; Sound Recorder).

#### 2.1.2. Study Target Population

The study population is residents (over the age of 21) of U.S. households who are exposed to relevant levels of nighttime air traffic (both in terms of number and *L*_AS,max_ of events) in communities in close proximity to an airport who meet the eligibility criteria for this study.

#### 2.1.3. Airport Selection and Study Simulation

For every hour of the day in 2018, the FAA provided the number of arrivals and departures for every runway at every airport in the contiguous U.S. plus Alaska, Guam, and Hawaii. Eligible airports were selected based on the average number of aircraft movements per hour in the period 10:00 p.m.–6:59 a.m. (definition of nighttime for calculating DNL in the U.S.). To account for the fact that similar geographic regions are flown over for arrivals on a given runway and departures from the opposite runway (e.g., arrival to Runway 14 or a departure from Runway 32), runways were grouped into “runway ends”. Runway ends that averaged less than one movement per hour during the DNL night period were excluded. Following this procedure, 666 runway ends at 111 airports remained with ca. 1.4 million records of data.

For each of these 666 runway ends, we simulated the NSS. For 50 out of 52 weeks (excluding the weeks of Thanksgiving and Christmas), we simulated ten subjects living under the flight path of each runway end (i.e., 500 simulations per runway end total). We randomly drew a subject from the ATL and PHL pilot studies and used the observed sleep period times of that subject for our simulations, randomly drawing a sleep period time from the selected subject (with replacement) for five nights total. Median sleep period time was 7.45 h (range 3.03–11.8 h, interquartile range 6.58–8.16 h) in the ATL and PHL pilot studies, while median sleep onset and sleep offset were 11:08 p.m. (range 7:53 p.m.–5:15 a.m., interquartile range 10:21 p.m.–11:56 p.m.) and 6:35 a.m. (range 2:30 a.m.–11:43 a.m., interquartile range 5:44 a.m.–7:13 a.m.), respectively. We counted the number of ANEs to which this simulated subject would have been exposed during the five nights. Raw data and percentiles for observed number of events were stored by runway end.

Runway ends were further divided into three classes based on the criteria outlined below and cutoff values were chosen on the following rationale:

- Awakening probability attributable to noise at the highest noise levels experienced in the bedroom is typically around 10%. Airports in Germany (Leipzig–Halle) [17] and Canada (Montreal) [27] use one additional awakening induced by nighttime aircraft noise per night as a criterion for limiting or assessing the effects of aircraft noise on sleep. Thus, minimally 10 events per night (50 events per five nights) are needed to reach this threshold. We used the median number of observed aircraft noise events during the five simulated nights for classification.
- For statistical efficiency, instances in which investigated subjects are not exposed to a single aircraft noise event throughout the whole measurement period should be rare in the actual study. Runway ends where >5% of subjects had zero observed events were classified as low-traffic runway ends.

Accordingly, runway ends were categorized into the following three classes based on the number of aircraft noise events (ANEs) observed across all five simulated nights:

- **Low-Traffic Runway Ends**: 0 ANEs in the 5th percentile or median of less than 50 ANEs (464 runway ends fell into this category; all runway ends were classified as low traffic at 34 airports)
- **Medium-Traffic Runway Ends**: More than 0 ANEs in the 5th percentile and a median of 50–99 ANEs (119 runway ends fell into this category; at least one runway end was classified as medium traffic at 44 airports)
- **High-Traffic Runway Ends**: More than 0 ANEs in the 5th percentile and a median of at least 100 ANEs (83 runway ends fell into this category; at least one runway end was classified as high traffic at 33 airports)

### 2.2. Sample Size and Power Calculations

#### 2.2.1. Primary Outcome and Assumptions

The primary outcome of aircraft noise-induced awakening probability is obtained using an estimated exposure–response function that describes the relationship between the *L*_AS,max_ of an aircraft overflight measured inside the bedroom and the probability of awakening determined by changes in heart rate and body movement.

During data analysis, a certain window after the start of an aircraft noise event is screened for an awakening from sleep. The duration of this window is critical (see Brink et al. [28]). If the window is too short, noise-induced awakenings may be missed. If it is too long, awakenings unrelated to aircraft noise may be picked up. For the analyses presented below, we used a screening window from 5 s before until 45 s after the start of an ANE, for a total length of 50 s for each noise event. These durations were shown to maximize signal-to-noise ratio in the pilot studies [23,24]. The screening window is extended to five seconds prior to the start of the ANE to account for uncertainties in the correction of the relative time drift between the sound recorder and the Faros ECG monitor (see Section 2.4.2).

The target sample size was determined using simulation studies for the number needed to estimate P(Awake|*L*_AS,max_ = 50 dB) − P(Awake|*L*_AS,max_ = 30 dB) with a 95% confidence interval half-width no larger than 0.015. This interval width was considered a priori to be an acceptable level of precision of the exposure-response function for regulatory purposes. The quantity P(Awake|*L*_AS,max_ = 50 dB) − P(Awake|*L*_AS,max_ = 30 dB) describes an attributable risk, i.e., the risk of waking to aircraft noise above and beyond the risk to wake spontaneously (i.e., for reasons other than aircraft noise [28]), as awakenings are not specific for aircraft noise, i.e., a participant may wake for reasons other than aircraft noise (e.g., to change body position or due to a dream). The quantity P(Awake|*L*_AS,max_ = 50 dB) − P(Awake|*L*_AS,max_ = 30 dB) was chosen for the target precision because it represents the excess probability of awakening for an ANE in the upper range of the expected aircraft noise levels in the bedroom (approximately 75% of the expected aircraft *L*_AS,max_ will be less than 50 dB) compared to a noise level equaling the average expected background level in the bedroom (30 dB *L*_AS,max_) [24]. The width of the 95% confidence interval for an *L*_AS,max_ less than 50 dB will also be controlled; this is due to the combined factors that, for increasing exposure levels, the estimated probability will become more variable as it moves away from zero (and stays less than one-half for the expected observed range of *L*_AS,max_), and the increased uncertainty at the extreme levels of exposure of a fitted regression for the linear predictor on the logit scale [29].

#### 2.2.2. Simulation Studies to Determine Statistical Power

Pilot data from four studies (see Appendix B) were used to inform parameters used in simulations to assess the uncertainty of the primary outcome at different sample sizes. A series of models were run to derive these parameters. In Models 0, 1, and 2 shown below, β’s are the fixed effects for intercept and slope of *L*_AS,max_, and b’s are the random effects for intercept and slope at the individual (i) and airport (j) level. Model 0 was used to fit the pilot data to each airport (indexed by j) separately, where i indexes the individuals in the pilot study, and the average fitted fixed effect coefficients across the airports were considered the true fixed effect parameters for the simulation data-generating model, Model 2 (β_0_, β_1_).

Model 0: Logit(P(Awake)) = (β_0_ + b_0i_) +(β_1_ + b_1i_) *L*_AS,max_

The variance–covariance matrix for the airport-specific random effect terms (var(b_0j,_ b_1j_)) of the data-generating Model 2 are based on the between-airport variance of the (β_0,_ β_1_) across the four pilot study airports, as explained further below. Similarly, the person-specific random effect terms (b_0i_ and b_1i_) are informed by their average values across the four pilot models. Model 1 was used to fit the simulated data generated with Model 2. Each individual has their own baseline probability of awakening, and each airport has its own baseline probability of awakening and dose–response effect.

Model 1: Logit(P(Awake)) = (β_0_ + b_0i_ + b_0j_) +(β_1_ + b_1j_) *L*_AS,max_

The probability of waking for subject i at airport j with an aircraft *L*_AS,max_ in dB is expit((β_0_ + b_0i_ + b_0j_) +(β_1_ + b_1j_) *L*_AS,max_). Based on the pilot data, it is anticipated that this is the most complex model that will be reliably fit to the data, but that will be confirmed using goodness of fit statistics at the time of data analysis (see Section 3.3). Model 2 was the data generating model for the simulations used to estimate the required sample size.

Model 2: Logit(P(Awake)) = (β_0_ + b_0i_ + b_0j_) +(β_1_ + b_1i_+ b_1j_) *L*_AS,max_

Parameter estimates from Model 0 fit to the pilot data were used to estimate the true fixed effects, individual random effects, and airport random effects. Each individual and airport has their own baseline probability of awakening and dose–response in Model 2. Our primary outcome is β_1_ (dose response for *L*_AS,max_) for probability of awake (P(Awake)) for the population level. Using ANEs from the four pilot studies, we ran 1000 simulations for each scenario with choice of:

1. **Traffic class** (all airports, medium and high traffic airports only, high traffic airports only)
2. **Variance of random effects** (type 1: empirical average of all pilot studies, type 2: theoretically set person-specific variance inflation of 20%, type 3: theoretically set person-specific variance inflation of 50%)
3. **Sampling scheme** (uniform, population density by household units)
4. **Sample size** (250, 300, 350, 400, 500)

Detailed modeling steps and the simulation algorithm can be found in Appendix C. Simulations were performed for all airports, medium and high traffic airports only, and high traffic airports only (see Section 2.1.3). The expectation was that statistical power would decrease when including airports with lower nighttime air traffic, but it was not clear how statistical power would be affected in detail, as there could also be increases in power due to increasing the number of airports. In addition to using the person-specific variance empirically derived from the four pilot studies (type 1), the variance was inflated by 20% (type 2) and 50% (type 3) to account for a potential underestimation of person-specific variance by the pilot studies. In addition, for each simulation run, 30% of ANEs were randomly deleted for each subject, and 5% of subjects were randomly deleted prior to running models to account for missing data (e.g., due to equipment failures) and participant drop-out. We explored both uniform and population density-based sampling strategies (population density data were not available for low traffic airports). We investigated sample sizes ranging from 250 to 500 participants. The results of the simulations can be found in Table 1.

**Figure 1:**
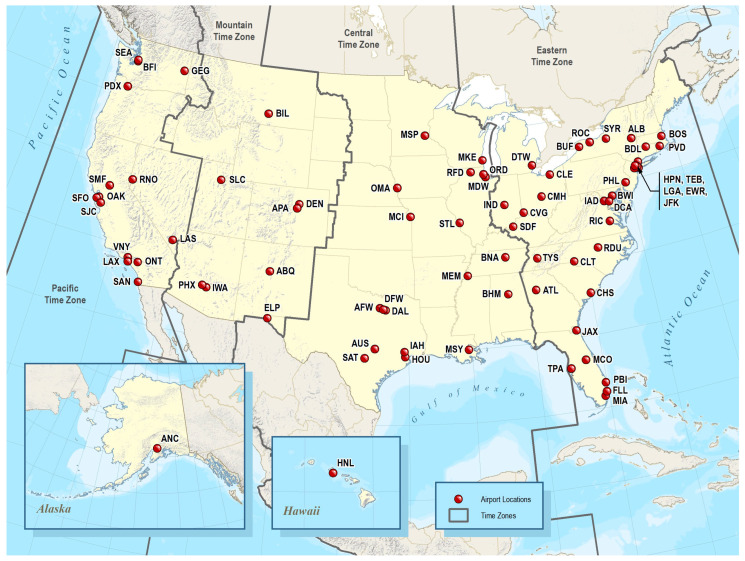
Map of International Air Transport Association’s location identifiers for the 77 medium and high traffic airports within the study sampling frame.

Simulations with uniform sampling and empirical random effect distribution showed a noticeable increase in confidence interval width if low traffic airports were included, which is why we decided to exclude low traffic airports from further simulations. Under the most conservative assumptions of a random effect distribution inflated by 50%, a sample size of N = 350 participants provided a confidence interval half-width just below the *a priori* defined threshold of 0.015 if only high traffic airports were included, and a sample size of N = 400 participants provided a confidence interval half-width just under 0.015 if only medium and high traffic airports were included. To increase the generalizability of the study, we decided to sample N = 400 participants at 77 airports classified as medium or high traffic. The selected 77 airports are shown in Figure 1.

As it was unclear to what extent traffic volumes changed due to the SARS-CoV-2 pandemic relative to 2018 traffic, the simulation studies were repeated by artificially reducing traffic in 10% increments up to 50%. These simulations indicated that the precision of the exposure–response function was not relevantly affected for traffic reductions up to 20%, and that sample size would need to be increased for more severe traffic reductions to maintain precision. It was decided that the traffic observed during the first year of the study would be compared to 2018 traffic, and that the sample size would be adjusted in the case of traffic reductions greater than 20%. This comparison indicated that traffic during the first study year in fact increased by approximately 28% relative to 2018, so no sample size adjustment was necessary.

### 2.3. Subject Enrollment

#### 2.3.1. Eligible Household Selection

**Figure 2:**
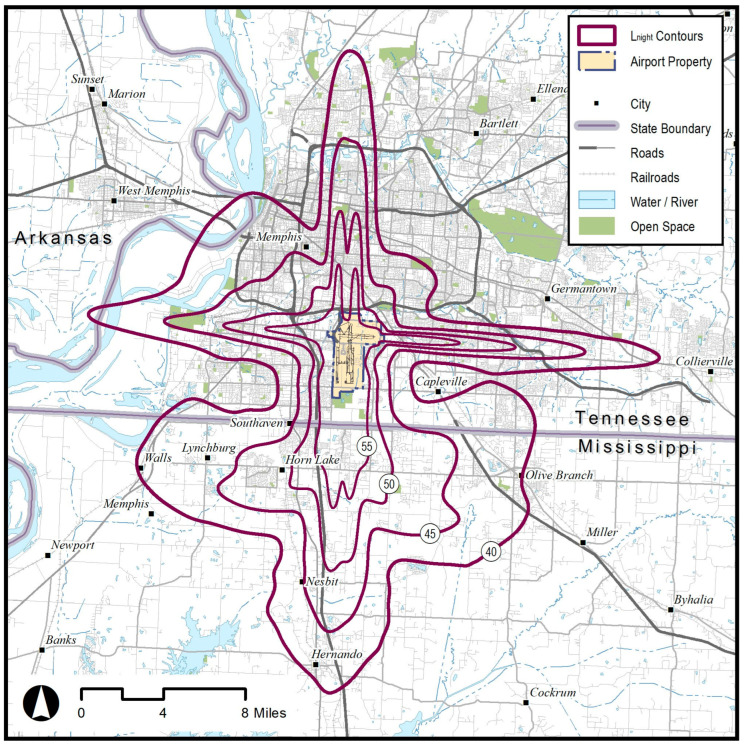
*L*_night_ contours used for stratified sampling are shown for Memphis Airport (MEM) as an example. Contours are based on 2018 traffic to and from runway ends that averaged at least one departure or arrival per hour between 10:00 p.m. and 6:59 a.m.

For each of the 77 selected medium or high traffic airports, the FAA generated 40, 45, 50, and 55 dB *L*_night_ contours based on 2018 traffic to and from the associated 202 runway ends that averaged at least one departure or arrival per hour between 10:00 p.m. and 6:59 a.m. (Figure 2). The shape (.shp) files of the contours were used to determine which census blocks were within (or partially within) the outermost noise contour (i.e., 40 dB *L*_night_). Residential addresses associated with the identified census blocks were obtained based on the Computerized Delivery Sequence File from the United States Postal Service. These addresses were geocoded to the appropriate noise strata. Geographic information systems (GIS) software was utilized to assign latitude and longitude to all addresses that fall within the outermost noise contour. Addresses falling outside of the outermost noise contour were excluded from the sampling frame (zero useable addresses were available within the noise contours of one airport). For the remaining addresses, we determined which of the four noise strata each address belonged to (i.e., 40 < 45, 45 < 50, 50 < 55, ≥55 dB *L*_night_) and 308 sampling cells were created by crossing the four noise strata with the 77 airports.

The sampling target for each sampling cell is proportional to the population within the sampling cell relative to the total population of all sampling cells in each noise stratum. A random sample of addresses is sampled within the 308 sampling cells based on the sampling targets identified (those around PHL and ATL who have already participated in the pilot study are ineligible to participate again in the NSS). Based on response rates observed in the pilot study around ATL, an 88% delivery rate for mailed Recruitment Surveys, a 21% response rate among deliverable surveys, and a 9% participation rate among completed surveys was expected. Thus, it was anticipated that 24,500 surveys needed to be mailed to recruit 400 participants into the in-home sleep study. For each of the four strata 6125 addresses were selected. In addition, a reserve sample of 19,980 addresses was sampled, for a total of 44,480 addresses, to allow for lower response rates than anticipated.

#### 2.3.2. Participant Recruitment

Due to the constraints of available in-home monitoring equipment and to investigate participants across the whole year, the sample is fielded over a 24-month period with a new randomly assigned subset of the sample released every month. Each round contains approximately 1/24 of the total sample with each noise stratum equally represented in each round. The first Recruitment Survey wave was sent out in September 2021 and the last wave was sent out in August 2023. Sample sizes are adjusted each month based on the accumulated response pattern data to achieve similar sample in-home study completion sizes in each of the four *L*_night_ strata.

The mailing protocol follows procedures outlined by Dillman et al. [30] which were tested in a previous pilot study [31]. The Recruitment Survey consists of 25 questions (see Appendix A) that were chosen to maximize response rates and minimize the burden on the respondent, while providing information necessary to determine field-study eligibility and generate sampling weights (see Section 2.3.3 and Section 3.3.4). All sampled addresses are contacted between 2 and 4 times, depending on when the questionnaire is returned. The materials are addressed to “<city> resident”. For the initial survey wave, respondents can only return the paper version of the survey in the pre-paid envelope. A thank-you/reminder postcard is sent out 7 days after the initial survey mailing. Only non-respondents to prior mail packages receive subsequent survey package mailings. Mailings returned as postal non-deliverable (PND) are excluded from subsequent mailings. If no response is received three weeks after the initial survey was sent out, a second Recruitment Survey is sent out. In the follow-up rounds, respondents can complete the survey either by mail or online; providing multiple response modes is an effective method to improve overall survey response [30]. If no response is received six weeks after the initial survey was mailed, a third and final Recruitment Survey is sent out. The survey mailing number (1, 2, or 3) will be indicated on the survey and represented in the dataset.

**Figure 3:**
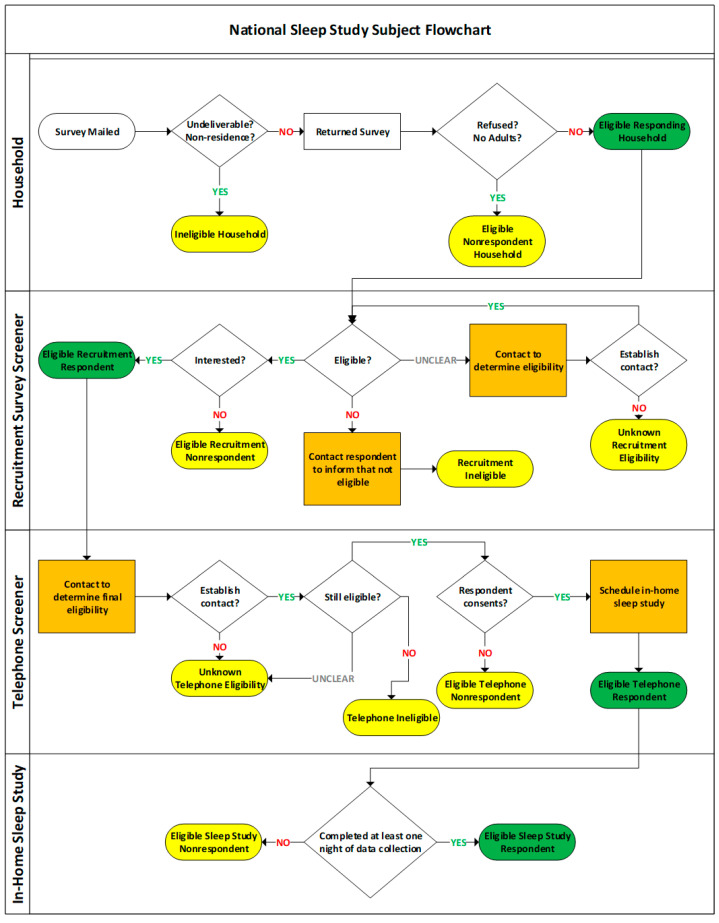
Field study participant enrollment process.

The contents of each survey packet include a cover letter, a brief overview of the field study, a paper questionnaire that the respondent is asked to return via an included postage-paid envelope or completed online (in the second and third mailing only), a USD 2 cash incentive (first packet only) [31], and a slip in Spanish that asks respondents who only speak Spanish to request a Spanish survey packet via telephone. A URL to a study web site at the University of Pennsylvania (UPenn) is provided for respondents who want to learn more about the field study, together with a telephone number by which the study team can be reached. One individual per household is randomly sampled to complete the survey using the Last Birthday Method. The instructions on the first page of the survey will ask the adult who most recently celebrated a birthday to fill out the questionnaire. Westat scans, verifies, cleans, harmonizes, and dispositions the paper and web Recruitment Survey data and securely delivers new respondent data to UPenn weekly. Survey respondents who indicated their interest in participating in the in-home study on the survey are contacted by the UPenn research team following the algorithm outlined in Figure 3.

If a respondent is both interested in participating in the study and meets the eligibility criteria (see Section 2.3.3), the respondent is contacted via telephone following standardized telephone scripts to verify eligibility and gain consent according to Institutional Review Board (IRB) regulations (if respondents do not provide a telephone number this will be solicited through either email or standard mail). There are minimal risks associated with taking part in the field study, which are described to interested respondents and explicitly stated in the informed consent form (ICF). Prospective participants are given ample time to read the ICF and contact the study team with any questions regarding the information provided in the ICF. If respondents decide to participate in the field study after review of the ICF, they can sign the ICF electronically. Alternatively, a paper version can be mailed by the study team. Once subject consent is received, staff members call to schedule the individual for participation in the field study (see Section 2.4).

#### 2.3.3. Eligibility Criteria

To be eligible to participate in the in-home study, participants must have completed the Recruitment Survey. Survey respondents interested in participating in the in-home sleep study are ineligible to participate if they (1) are less than 21 years of age (Recruitment Surveys received from individuals under 21 years of age are discarded); (2) have a Body Mass Index (BMI; calculated based on self-reported height and weight information) greater than 35 kgm^−2^ or less than 17 kgm^−2^; (3) have been diagnosed with a sleep disorder, including obstructive or central sleep apnea, narcolepsy, restless legs syndrome, or periodic limb movement syndrome; (4) frequently (3 or more times per week) use prescription or over the counter medication to aid sleep; (5) have a hearing impairment; (6) have a cardiac arrhythmia; (7) work night shifts (defined as working for at least 4 h between 12 a.m. to 6 a.m.); (8) have dependents that frequently require care during the night; (9) are pregnant; (10) participated in the ATL and PHL pilot studies; or (11) do not habitually use earplugs or play back sounds in the bedroom that could mask aircraft noise.

### 2.4. Field Study

The research team at UPenn is responsible for performing the field study, which is conducted year-round except for the week of Thanksgiving, the week of Christmas, and the weeks preceding and following Christmas (i.e., for 48 weeks of the year). On average, four participants are investigated per week to meet the total recruitment goal of N = 400 participants over a two-year period; although, more or fewer subjects may be investigated in any given week based on participant availability. The first participants were investigated in early October 2021 and the field study is expected to end in October 2023. Subjects participate in the field study for five consecutive nights/mornings starting on a Monday night and ending on the following Saturday morning. The weekend period was excluded as traffic volume typically decreases on weekend nights.

#### 2.4.1. Field Study Data Collection Procedures

A sound recorder, an ECG and movement monitor, and a printed manual are mailed to participants in the week prior to their scheduled start date. The manual contains (1) detailed instructions for setting up, breaking down, and mailing back the equipment; (2) step-by-step procedures to follow each night and morning of the study; (3) a Characteristics Questionnaire to be filled out once prior to the first study night (see below); and (4) Morning Surveys to be filled out each morning after waking. The manual also contains links to instructional videos with step-by-step explanations of equipment setup and study procedures.

Participants are instructed to adhere to their usual bedtimes. During the measurement period, they start and stop acoustic and physiologic measurements before they go to bed and after they get up, respectively. Sounds, ECG, and body movements are continuously recorded during this measurement period. Participants are instructed to turn off any TVs, radios, music, or sound machines before going to bed and not to wear ear plugs or headphones during the night. They are told to leave central or window air conditioning units or fans that keep them comfortable during the night on, but to place moveable fans as far away from the sound recorder as possible.

Each morning after waking the participants complete the Morning Survey, which is embedded in the manual sent to participants. The Morning Survey has questions on alcohol and caffeine consumption in the 6 h pre-bed; how stressful the last day was; whether the bedroom window was closed, partially open, or completely open during the night; whether the bed was shared; lights out time, wake time, rise time, sleep latency (defined as the time it takes to fall asleep), and the number of awakenings; tiredness, sleep quality, and sleep disturbance due to aircraft noise. It also assesses sleepiness with the Karolinska Sleepiness Scale [32] and allows participants to leave general comments at the end. It takes less than five minutes to complete the Morning Survey. After completing the Morning Survey, participants go about their normal daily and evening activities. Subjects can contact the study team through email and/or phone. Staff members are available around the clock via cell phone to answer any questions or concerns about equipment or procedures. The research team always calls participants on the first night and last day of their measurement period to answer any questions.

Participants also fill out the Characteristics Questionnaire prior to the first study night. The purpose of this questionnaire is to obtain additional information on field study participants that may affect or explain how they physiologically respond to the aircraft noise exposure. The Characteristics Questionnaire takes approximately 15 min to complete and includes:

1. Morningness–Eveningness Questionnaire (MEQ), a standardized method of measuring chronotype [33];
2. Pittsburgh Sleep Quality Index (PSQI), a standardized method of measuring sleep quality over the past month [34];
3. Noise Sensitivity Questionnaire (NoiseQ), reduced version, a standardized method for measuring sensitivity to noise [35,36]; and
4. Additional questions considered relevant but not included in the Recruitment Survey. The questions address noise countermeasures; sleep disturbance by road, rail, industry, construction, neighbors, and air conditioning over the past 12 months; whether the residence received sound proofing treatment; and whether participants use air conditioning in their bedroom. Questions on sleep disturbance are formulated according to standard guidelines described by the International Commission on the Biological Effects of Noise (ICBEN) [37].

After the last study night, participants follow instructions provided in the manual for breaking down and packing away the equipment and the manual, which they then ship back to the research team. After receiving the equipment at UPenn, participant data are backed-up and the equipment is cleaned, disinfected, tested for nominal operation, and then prepared for the next participant by the research team.

#### 2.4.2. Field Study Equipment

**Figure 4:**
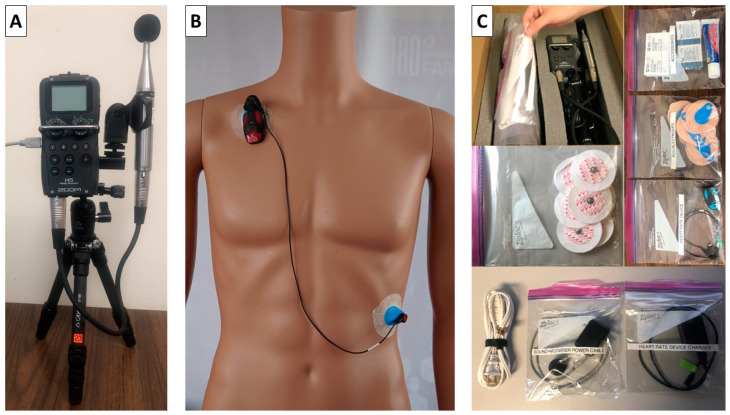
Field study equipment. (**A**) Sound recorder Zoom H5; (**B**) ECG and body movement device Bittium Faros 180; (**C**) illustration of all equipment components and how they are stored and packed for shipping.

The sound recorder and ECG/movement device are the main field study components. The materials sent to study participants also include protective foam, the manual, chargers and an extension cord, ECG electrodes (electrodes from two different brands are included in case a participant has an allergic reaction to the standard electrodes), disinfectant and adhesive removal wipes, as well as a corticosteroid cream, for skin preparation/care, tape and a return shipping label, and a payment form and Debit card (ClinCard). Most of these components are stored in labeled Ziploc bags (Figure 4C).

##### Sound Recorder

The H5 Handy Recorder (Zoom Corp, Tokyo, Japan) with an Earthworks M23 measurement microphone (Earthworks Inc., Milford, NH, USA) and windscreen is used for recording sounds in participants’ bedrooms (Figure 4A). Data are stored internally on an SD card in .mp3 format (320 kbps) and downloaded onto a secure, password protected server after completion of the study. A-weighted sound pressure levels (time-varying *L*_AS_) acquired with this setup agreed to within 1.5 dB compared to measurements with class-1 Sound Level Meters (SLM) over the frequency range of air traffic noise (see Section 3.1 of the Supplement of Smith et al. [24]). A 1 kHz calibration signal at 94 dB (Larson Davis CAL200) is recorded prior to sending out the equipment and after receiving it back. Data from participants where the absolute difference between pre- and post-study calibrations exceeds 1 dB are discarded. The pre-study calibration signal is also used to convert the recorded sounds into A-weighted sound levels (*L*_AS_ and *L*_AF_ at a 0.1 s resolution). After the equipment is returned by study participants, the microphone is placed 2 m in front of a Neumann KH 310 active studio speaker (Neumann, Berlin, Germany) and its frequency response is tested with a pink noise generator (NTi Minirator MR-PrO, NTi Audio, Schaan, Liechtenstein) using a class-1 SLM (NTi XL2 Analyzer). Microphones are replaced if they do not meet pre-specified criteria. All acoustic equipment (calibrators, microphones, class-1 SLM) is sent to the manufacturer for re-calibration at intervals specified by the manufacturer.

Before the first study night, participants are instructed to set up the sound recorder on their nightstand, at least five inches from any wall and near pillow position. If they do not have a nightstand, they can instead use the box in which the equipment was shipped. Participants record where they placed the sound recorder (nightstand, box, other). They keep the sound recorder plugged into an electrical outlet (the recorder does have battery backup) and the power turned on constantly throughout the study. They start a measurement before going to bed and stop it after getting up in the morning.

##### ECG and Body Movement Monitor

ECG and body movement are measured using the battery-powered cardiac monitor Faros 180 (Bittium Corp, Oulu, Finland, Figure 4C). The device can measure a single-lead ECG (Einthoven II) with a sample rate of up to 1 kHz and body movement using a 3-axis accelerometer with a sample rate of up to 100 Hz and 14 bit resolution. Different sample rate settings were tested prior to the study as sample rate affects both data storage requirements and battery life. Based on these tests, the optimal sampling rates were 250 Hz for ECG and 25 Hz for body movement (with a dynamic range of 2 g and 14 bit resolution) as this assured that the device’s battery will not deplete even if a participant never charges the device during the 5-night study. Furthermore, a 250 Hz ECG sample rate permits frequency–domain heart rate variability analysis [38], which is a relevant secondary outcome that estimates the parasympathetic regulation of cardiac function. Respiratory movements can be inferred from the accelerometer signals and provide data suggestive of sleep apnea (see Section 3.1.2).

During measurement nights and after skin preparation, participants are asked to place one electrode just below the right clavicle and another below the left breast (Figure 4B). The device turns on automatically once the electrodes are connected to the chest. In the morning after getting up, participants are instructed to put the Faros device on the charger, which triggers the device to stop the measurement automatically. Participants are instructed to change the placement of the ECG electrodes slightly each night to avoid skin irritability or allergic skin reactions.

##### Time Synchronization

Prior to the start of the study, it was established, in experiments simulating the NSS, that the internal clocks of both the sound recorders and the Faros devices drift in time relative to atomic clock time (see Section 3.2 of the Supplement of Smith et al. [24]). The Faros devices consistently ran faster by an average of 0.7 s per day (range 0.2–1.2 s per day) relative to atomic clock time. The sound recorders ran either faster or slower by an average of 0.2 s per day (range 1.2 s per day slower to 1.4 s per day faster) relative to atomic clock time. Sound recorders and Faros 180 devices were paired into one of 30 study kits in a way that the relative drift between devices was minimized. To facilitate event-related analyses, study staff perform a time synchronization procedure before the equipment is mailed to the participant and upon receipt at UPenn from study participants. The time offset relative to atomic clock time is noted and used to calculate the device time drift. As each device is used multiple times throughout the study, each device’s drift will be well characterized by the end of the study. Time stamps are corrected during data analysis (see Section 3) so that all times reflect atomic clock time. Finally, participants perform a time synchronization procedure each night right after starting measurement on both devices. They are instructed to tap the Faros device five times while saying out loud “1, 2, 3, 4, 5” with each tap. The tap and the spoken numbers can easily be identified in the data streams and used as further data points for time synchronization.

#### 2.4.3. Incentives

There are three types of incentives for participants in the study: an incentive for completing the Recruitment Survey, an incentive to take part in the field study, and incentives for providing additional self-report data during participation in the field study.

A USD 2 cash incentive is included in the first mailing of the Recruitment Survey questionnaire package to encourage responses from selected households. Pre-paid incentives of this size have been shown to increase response to postal surveys significantly [30,39,40]. Furthermore, in a pilot study, a USD 2 cash incentive almost tripled the response rate compared to promised gift cards of USD 2, USD 5, or USD 10 value [31].

Subjects who participate in the in-home sleep study are paid a maximum of USD 170 for the 5-night study: USD 30 for each night they complete the sleep and noise measurements (a total of USD 150 if recorded each night), USD 2 for each completed Morning Survey (a total of USD 10 if the questionnaire is completed each morning), and USD 10 for completing the one-time Characteristics Questionnaire.

To receive payment for their participation, participants must complete payment forms and return the study equipment and questionnaires. A waiver from collecting social security numbers (SSN) for the purpose of subject payments from UPenn was obtained; therefore, SSN information from the participant is not necessary for payment. The equipment sent to participants includes a pre-paid Debit card (ClinCard). Funds on this card are activated after the equipment is returned to UPenn and it is verified that data were recorded. If a participant decides to withdraw from the study before the study is over, they are fully compensated for their participation up to the point at which they chose to withdraw.

#### 2.4.4. Ethics

There is minimal risk associated with the recruitment or field study questionnaires. Participants’ names and addresses specifying where to mail the Recruitment Surveys were obtained by Westat. Participants also provide their email and phone number on the survey if they want to take part in the in-home sleep study. All study related data that contain personally identifiable information (PII) are stored on secure servers with limited personnel access and passwords or in locked cabinets. There is a possible risk of a loss of confidentiality and privacy in rare circumstances.

For participants in the in-home sleep study, measuring heart activity (ECG) and body movement also involves minimal risks. The Faros device is powered by batteries that pose no risk to participants, although the device itself may cause minor discomfort during sleep. The electrodes attached to the chest may cause some minor discomfort and skin irritation.

There is minimal risk associated with recording indoor sounds. The sound recordings are listened to by staff to identify the source of noise events during the sleep period. There is a possible risk of a loss of confidentiality and privacy in very rare circumstances. However, the indoor sound recordings are only made at night, and the subjects will start and stop the indoor sound recording equipment each night themselves. The recordings are stored on a secure server with limited personnel access and passwords. The files are named using study codes. If any identifiers are detected by staff during analysis, this information is removed from the recordings.

The study was approved by the IRB of the University of Pennsylvania (protocol #833863) and by Westat IRB (protocol #6739). The study was also approved by the Office of Management and Budget (OMB Control #2120-0798). The study was registered at clinicaltrials.gov under NCT05035940. Subjects gave written informed consent prior to participation in the field study and were able to discontinue participation at any time without providing reasons.

## 3. Data Analysis

### 3.1. Processing of Returned Equipment and Field Study Data

Research coordinators process the equipment returned by field study participants, using checklists, as soon as possible after delivery. The equipment is checked for integrity, cleaned, and disinfected. Unused consumables (e.g., ECG electrodes) are discarded, and the shipping box is replaced. Post-study timestamps are recorded for the sound recorder and the Faros device and put into a participant-specific time synchronization database. The sound files for each of the five study nights are transferred to a secure server. Pre- and post-study calibration values are compared with software developed in MATLAB (version R2020b, The MathWorks Inc., Natick, MA, USA). Study night .mp3 files are automatically converted to sound levels (see Section Sound Recorder) with a batch version of the same software. Microphones are tested and replaced if necessary (see Section Sound Recorder). Faros ECG files for each of the five study nights are also transferred to a secure server. Participant time-synchronization taps and spoken words are identified at the beginning of each night and later entered into a RedCAP study log. Surveys completed by participants during the field study are scanned and saved as PDFs on the server before they are entered manually into a RedCAP database. Except for open questions, research coordinators must choose from pre-defined answer categories to minimize data entry errors.

#### 3.1.1. Processing of ECG and Movement Data

Clinical research coordinators inspect the ECG signal of the first study night with an EDF viewer, and note the number and type of irregular heartbeats, if any, and alert the principal investigator who then notifies study participants if necessary.

A software developed in MATLAB is used to display respiratory movements. Clinical research coordinators inspect respiratory patterns on the first two study nights and alert the principal investigator if they observe patterns indicative of obstructive or central sleep apnea, in which case the principal investigator notifies study participants if necessary.

A validated software developed in MATLAB is then used to automatically detect EEG awakenings based on body movement and changes in heart rate (see [22] for details). Clinical research coordinators inspect the data and mark periods of ECG signal loss (e.g., caused by a loose or fully detached electrode) or ECG artifacts following standard operating procedures. During these periods, the algorithm detects awakenings from sleep based on body movement only, which is slightly less accurate but still considered “almost-perfect” according to conventional standards [22]. It is recorded, for subsequent statistical analyses, whether an awakening was detected based on body movement only, or based on both changes in heart rate and body movement.

#### 3.1.2. Processing of Sound Recorder Data

Clinical research coordinators listen to the sounds recorded by field study participants and mark the beginning and the end of each ANE in a software developed in MATLAB following standard operating procedures. Primary and secondary noise events are differentiated during this process. An ANE is only classified as primary if it generates the highest noise level during the event period, otherwise, it is classified as secondary. There is one exception to this rule: ANEs that induce or coincide with a movement or awakening of the participant that generate a higher sound level than the ANE, which are nevertheless scored as primary events. Excluding these events would bias the exposure–response function to shallower slopes. In addition to ANEs and secondary events co-occurring with them, research coordinators mark periods when participants get out of bed (e.g., for bathroom visits). These periods are not included in data analysis, as participants were apparently awake. The software automatically identifies background noise levels in 10 min intervals throughout the night. This automatic detection is checked by research coordinators and corrected if necessary.

To facilitate the identification of aircraft noise events and to increase objectivity and reduce bias for this processing step, radar flight-track data provided by the FAA are used by HMMH to determine the time of point of closest approach (POCA) to (the address of) each respondent. The radar data provides aircraft flight tracks, with each track consisting of aircraft coordinates spaced every four seconds or so. The three-dimensional slant distance between the respondent (five feet above the ground) and the aircraft is computed for each pair of coordinates, interpolating between segment endpoints if necessary. The minimum slant distance is the POCA for that track and the time associated with the POCA is computed. In addition to the time of POCA, HMMH provides, amongst others, the slant distance of the aircraft to the respondent at the time of POCA. The speed of sound traveling through the atmosphere depends on several variables, of which air temperature is the most relevant. Ambient air temperature data are extracted for each airport location in hourly intervals and used together with the slant distance to estimate the time needed for the sound emitted from the aircraft to travel to the respondent. The time of POCA is corrected accordingly. It is further corrected for any time drift of the sound recorder relative to atomic clock time. In addition to the corrected time of POCA, research coordinators are provided with a categorization of the slant distance of the aircraft: very close (less than 5641 ft); close (between 5641 ft and 13,281 ft); far (between 13,281 ft and 28,093 ft); and very far (at least 28,093 ft). The cut-offs were informed by quartiles of the slant distance distribution found at the 2021 field respondents.

After research coordinators are finished with marking ANEs, the software automatically generates a list of noise events that includes several acoustic-noise-event descriptors including *L*_AS,max_ and the background noise level (*L*_A,eq_) one minute prior to the start of the noise event. This list is later corrected for time drift and used for event-related analyses (see Section 3.3). The software also generates a file that includes several acoustic descriptors of the sleep period (e.g., *L*_A,eq_ for ANEs, number of ANEs above certain thresholds). These are used for analyses of variables that reflect the whole night (e.g., Morning Survey responses; see Section 3.3).

### 3.2. Aircraft Noise Exposure Metrics

Using FAA radar data covering 2021 to 2023, HMMH will use the FAA’s Aviation Environmental Design Tool (AEDT) to compute the A-weighted Sound Exposure Level (*L*_AE_) and *L*_A,max_ for each flight track. These single-event metrics will allow UPenn/Westat to compute a multitude of cumulative event metrics such as DNL, *L*_night_, etc., for various bases (e.g., calendar year, month, week of testing, etc.).

### 3.3. Statistical Analysis

All statistical analyses will be performed with SAS (SAS Institute, Carey, NC, USA) or R (R Core team, Vienna, Austria), as appropriate. A type-I error probability of 0.05 is used to determine statistical significance.

#### 3.3.1. Primary Outcome

The probability of awakening from sleep (assessed with heart rate and body movement) depending on the aircraft *L*_AS,max_ (measured inside the bedroom) constitutes the primary endpoint of this study. A generalized linear mixed effects model (Model 1) will be fit to the field study data to estimate the exposure–response function for the *L*_AS,max_ of an ANE and awakening probability. Awakening probability at background noise level will be subtracted from the predicted awakening probability at higher noise levels to account for spontaneous (i.e., non-noise related) awakenings. The use of sampling weights to account for selection bias will be explored (see Section 3.3.4). Models may be additionally adjusted for acoustic (e.g., background noise level), situational (e.g., elapsed sleep time), and sociodemographic (e.g., age, sex, SES) variables if appropriate. Standard model diagnostics will be used to assess whether the random effects included in the model adequately capture the clustering, or whether the full Model 2 or a model with fewer random effects offers a superior fit.

#### 3.3.2. Secondary Outcome

The Recruitment Survey contains the question: “Thinking about the last 12 months or so, when you are here at home, how much does noise from aircraft disturb your sleep?” Subjects choosing the two most extreme responses (“very” or “extremely”) on the 5-point answer scale will be considered highly sleep disturbed. The percentage of subjects that are highly sleep disturbed constitutes the secondary outcome of the NSS. Associations between DNL and *L*_night_ (see Section 3.2) and the odds of being highly sleep disturbed will be investigated in logistic regression models. As with the primary outcome, we will explore the use of sampling weights specifically generated for Recruitment Survey respondents (see Section 3.3.4). We will also consider adjustment for additional acoustic, situational or sociodemographic variables.

#### 3.3.3. Exploratory Analyses

Acoustic descriptors of the sleep period (e.g., *L*_A,eq_, number above threshold) will be associated with physiologic descriptors of the sleep period (e.g., sleep efficiency, sleep fragmentation index) as well as subjective ratings provided by study participants in the Morning Surveys (e.g., sleep quality). Models using acoustic descriptors measured inside the bedroom will be compared to models using acoustic descriptors predicted based on traffic data on the outside of the dwelling.

In addition to sleep disturbance (secondary outcome), we will investigate how DNL, *L*_night_, and potentially other long-term acoustic descriptors are associated with other outcomes collected in the Recruitment Survey (i.e., sleep quality; sleep medication use; aircraft noise annoyance; neighborhood quality; health; hypertension; arrythmia; heart disease; diabetes; cancer). All models will use sampling weights and adjust for confounders where appropriate. Appropriate corrections for multiple testing will be applied in the exploratory analyses.

An analysis of differences between field study participants and non-participants based on the Recruitment Survey’s demographic questions will also be performed.

#### 3.3.4. Weighting

The weight for each enrolled participant is calculated based on the response outcomes from the four steps: (1) the response to the Recruitment Survey of the randomly selected address in a noise stratum of an airport; (2) the person level response to the Recruitment Survey (willingness to participate and eligibility); (3) the response to the phone screening questions; and (4) the data availability from the recording equipment in the field study. For each of the four steps, the weight is first adjusted for unknown eligibility, then for eligible but nonresponding persons/addresses. Eligible-unknown and nonresponding persons/addresses are treated as randomly missing. The sum of the final weight should be equal to the total number of potentially eligible persons within each of the survey strata. Based on the answers to the first stage Recruitment Survey, a household base weight for each sampled address is the reciprocal of the address’s selection probability. The address selection probability is the number of sampled addresses within a noise stratum divided by number of addresses in the noise stratum. The household base weight is adjusted for not responding, missing key data elements (number of age-eligible persons in household), or no eligible persons in the household. The sum of the adjusted household weights is the same as the sum of the base weight of the respondent and nonrespondents. To represent the number of age-eligible persons, the recruitment weight is created from multiplying the adjusted household weight and the number of age-eligible persons in the household. The recruitment weight is then adjusted first for unknown eligibility then for eligible but nonresponding persons. Based on the answers to the phone call screening questions, the adjusted recruitment weight is further adjusted first for eligibility-unknown persons, then for eligible but nonresponding persons to create a telephone screener weight. After the study equipment is sent to the respondents eligible for this study, the final sleep study weight is computed by adjusting telephone screener weights of responding persons for persons who do not collect data for at least one night.

## 4. Discussion

This manuscript describes the design, procedures, and analytical approach of the FAA’s National Sleep Study (NSS), which were informed by two pilot field studies conducted at PHL and ATL as well as by two field studies performed by the German Aerospace Center (DLR) around the Cologne Bonn (STRAIN study) and Frankfurt airports (NORAH study) [17,23,24,41]. Seventy-seven U.S. airports with relevant nighttime air traffic from 39 states are included in the sampling frame. Based on simulation-based power calculations, the study aims to recruit 400 participants from 24,500 Recruitment Surveys into a field study in which they record ECG, body movement, and sound levels in the bedroom for five consecutive nights year-round. To our knowledge, this is the most comprehensive study to date investigating the effects of aircraft noise on sleep.

The primary outcome of the study is an exposure–response function between *L*_AS,max_ generated by individual aircraft and measured in the bedroom, and awakening probability inferred from changes in heart rate and body movement. Self-reported high sleep disturbance due to aircraft noise is the secondary outcome that will be associated with the long-term noise exposure metrics DNL and *L*_night_. The effect of aircraft noise on several other physiological and self-report outcomes will be explored as well.

At the time of writing (September 2023), the study has successfully collected field study data from 385 participants. Data acquisition is expected to conclude by the end of October 2023. The data acquisition period will be followed by a two-year period of data analysis. A final report for the study is expected in 2025.

## 5. Conclusions

The FAA’s National Sleep Study is designed to provide large-scale, objective, physiological data on the effects of aircraft noise on sleep in the United States, which can be used by the FAA to inform the effectiveness of current noise regulation. The study is unprecedented in its size and scope and is poised to become a landmark study for the understanding of the effects of aircraft noise on sleep. The study was conducted by an interdisciplinary team with extensive experience in noise-effects research, statistical data analysis, noise modeling, and survey design and analysis, which increases the likelihood of its success.

## Figures and Tables

**Table 1:** Simulation study results. Values represent the half-width of the 95% confidence interval of P(Awake|*L*_AS,max_ = 50) − P(Awake|*L*_AS,max_ = 30).

Traffic	Sampling	Variance	Sample Size				
			250	300	350	400	500
L, M, H	U	1	0.0197	0.0189	0.0166	0.0161	0.0146
M, H	U	1	0.0149	0.0138	0.0127	0.0125	0.0106
H	U	1	0.0142	0.0132	0.0127	0.0113	0.0109
M, H	P	1	0.0153	0.0146	0.0132	0.0127	0.0111
H	P	1	0.0147	0.0145	0.0131	0.0124	0.0119
M, H	P	2	0.0161	0.0147	0.0142	0.0135	0.0121
H	P	2	0.0155	0.0151	0.0140	0.0139	0.0123
M, H	P	3	0.0168	0.0158	0.0151	**0.0143**	0.0129
H	P	3	0.0176	0.0162	0.0149	0.0149	0.0131

Traffic: airports included in the simulation (L = low traffic, M = medium traffic, H = high traffic); Sampling: uniform (U) or population density (P); Variance: random effect distribution (1 = empirical; 2 = 20% inflation; 3 = 50% inflation). The number in bold font informed the required sample size of 400 assuming medium and high traffic airports, population density sampling and 50% variance inflation.

**Figure A1:**
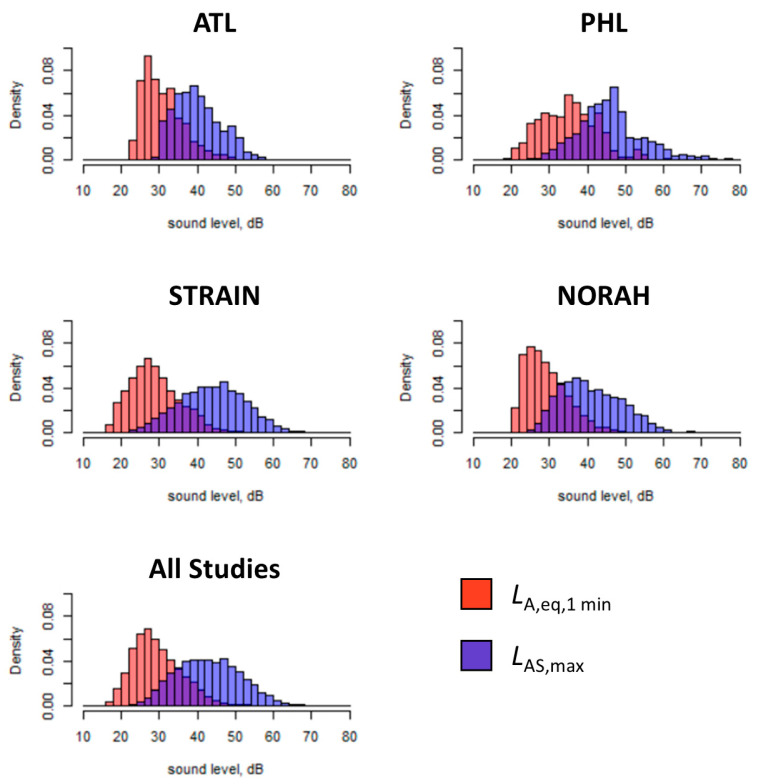
Frequency distribution for maximum sound pressure levels (*L*_AS,max_) and background noise levels in the period one minute prior to the start of the aircraft noise event (*L*_A,eq,1 min_) for the four field studies at Atlanta (ATL), Philadelphia (PHL), Cologne Bonn (STRAIN), and Frankfurt (NORAH) airport that were used to inform power calculations.

## Data Availability

At the end of the study, datasets will be de-identified and submitted by UPenn and Westat to the FAA. The FAA may decide to make (part of) the de-identified data publicly available for other entities to use.

## Appendix A

The Recruitment Survey includes the following questions:

Q1. *During the last month or so, how would you rate your sleep quality overall?*
  5-point Likert scale with answer categories very good; fairly good; neither good nor bad; fairly bad; very bad
Q2. *Select the response that best reflects how often you have taken medicine (prescribed or “over the counter”) to help you sleep during the last month or so.*
  4-point Likert scale with answer categories not during the past month; less than once a week; once or twice a week; three or more times a week
Q3. *How strongly do you agree or disagree with the statement “I am sensitive to noise”?*
  5-point Likert scale with answer categories strongly disagree; somewhat disagree; neither agree nor disagree; somewhat agree; strongly agree
Q4. *Thinking about the last 12 months or so, when you are here at home, how much does noise from aircraft bother, disturb or annoy you?*
  5-point Likert scale with answer categories not at all; slightly; moderately; very, extremely
Q5. *Thinking about the last 12 months or so, when you are here at home, how much does noise from aircraft disturb your sleep?*
  5-point Likert scale with answer categories not at all; slightly; moderately; very, extremely
Q6. *Now considering how you feel about everything in your neighborhood, how would you rate your neighborhood as a place to live on a scale from 1 to 5?*
  5-point Likert scale with anchors best (1) and worst (5)
Q7. *In general, would you say your health is…?*
  5-point Likert scale with answer categories excellent; very goof; good; fair; poor
Q8. *Have you ever been diagnosed by a health professional with any of the following sleep disorders? (Mark all that apply.)*
  Response options: sleep apnea; narcolepsy; restless legs syndrome; periodic limb movement syndrome; insomnia; none; other (please specify)
Q9. *Do you have any problems or difficulties with your sense of hearing?*
  Response options: yes; no
Q10. *Have you ever been diagnosed by a health professional with any of the following conditions? (Mark all that apply.)*
  Response options: hypertension/high blood pressure; arrhythmia/irregular heart beat; heart disease; diabetes; cancer; none
Q11. *What is your current employment status? (Mark one.)*
  Response options: employed (working mostly from home ); employed (working mostly away from home; unemployed/searching for a job; student; retired; homemaker; other
Q12. *What is the highest degree or level of school you have completed?*
  Response options: less than high school; high school graduate, including equivalency; some college credit, including Associate’s degree; Bachelor’s degree; graduate or professional degree
Q13. *What was your total household income last year?*
  Response options: less than $10,000; $10,000 to $14,999; $15,000 to $24,999; $25,000 to $34,999; $35,000 to $49,999; $50,000 to $74,999; $75,000 to $99,999; $100,000 to $149,999; $150,000 or more
Q14. *If currently employed, does your job require overnight shift work? (Overnight shift work refers to work for at least 4 h between 12 midnight to 6 a.m. in the morning.)*
  Response options: yes; no
Q15. *What is your ethnicity?*
  Response options: Hispanic or Latino; not Hispanic or Latino
Q16. *What is your race? (Mark all that apply.)*
  Response options: American Indian or Alaska Native; Asian; Black or African American; Native Hawaiian or Other Pacific Islander; White; prefer not to answer; other (please specify)
Q17. *How long have you lived at your current residence?*
  Response options: less than 1 year; 1 year or more but less than 5 years; 5 to 10 years; more than 10 years
Q18. *How many people 21 years of age or older (including yourself) reside in this household?*
  2-digit entry box for age
Q19. *Is there someone living in your home that frequently requires your care during the night?*
  Response options: yes; no
Q20. *What is your sex?*
  Response options: male; female
Q21. *What is your age?*
  2-digit entry box for age
Q22. *What is your height?*
  1-digit entry box for feet and 2-digit entry box for inches
Q23. *What is your weight?*
  3-digit entry box for pounds (lbs)
Q24. *Any other comments*
  Open question
Q25. *Are you interested in taking part in the in-home sleep study, and do you give your permission for the study team to contact you either by phone or email?*
  Response options: yes; no (input fields for first name, last name, email address, phone # [landline] and phone # [cell] are also provided).

## Appendix B

Data from four existing field studies on the effects of aircraft noise on sleep were used for power calculations. These studies were:

1. Study performed around Cologne Bonn airport (STRAIN) in 2001 and 2002 [17]
2. Study performed around Frankfurt airport (NORAH) in 2013 [41]
3. NSS pilot study performed around Philadelphia International Airport (PHL) in 2014 and 2015 [23]
4. NSS pilot study performed around Hartsfield–Jackson Atlanta International Airport (ATL) in 2016 and 2017 [24]

The number of investigated subjects and the number of noise events that contributed to data analysis are listed for each study in Table A1.

The distribution of maximum sound pressure levels (*L*_AS,max_) and background noise levels in the period one minute prior to the start of each aircraft noise event (*L*_A,eq,1 min_) for the four field studies that were used to inform power calculations and to simulate the NSS are shown in Figure A1.

**Table A1:** Overview of pilot studies used for power calculations.

Pilot	Number of ANEs	Number of Individuals
STRAIN	17,058	63
NORAH	8979	164
PHL	2010	32
ATL	1667	22
Total	29,714	282
Average	7428	70

## Appendix C

Modeling steps

1. Determine the fixed effects coefficients (beta = (β_0_, β_1_)) and airport-specific random effects
  - Fit Model 0 to ANE data from four pilot studies: ATL, PHL, STRAIN, NORAH. Each pilot study has a beta vector of (β_0_, β_1_).
  - The distribution of the beta coefficients across the four pilot studies is used to estimate the Model 2 parameters for the sampled airports in the simulation study. For the simulations, each airport will have a set of fixed effect coefficients for Model 2 drawn from a normal distribution, where the mean and covariance matrix are set to be the average value and covariance of those parameters across the four pilot studies.
  - The average beta parameter vector is (−4.2586, 0.0434), with estimated SD (0.6328, 0.0134) and covariance(beta) = −0.008430 defining the between-airport random effects. The variance for the (normal) distribution of beta parameters for individuals within an airport is determined by the random effects described in step 2.
2. Determine the individual random effects (b = (b0, b1))
  - Fit Model 0 to ANE data from four pilot studies: ATL, PHL, STRAIN, NORAH. Each pilot has random effects for intercept (b_0_) and slope (b_1_) for each individual. We define varRE to be the covariance matrix for individual random effects (b_0_,b_1_)
  - The true varRE used for simulations is calculated by using:
    - Type 1: the unweighted empirical average of varRE over all pilots The SD for (b_0_,b_1_) are (1.6376,0.0363), with covariance −0.0573.
    - Type 2: theoretically set variance inflated by 20% of type 1
    - Type 3: theoretically set variance inflated by 50% of type 1
3. *L_AS,max_ was left as a continuous variable and in its original scale.*

Simulation algorithm

The simulation algorithm given a fixed airport grouping, number of subjects, varRE type, choice of sampling scheme is as follows:

For simulation 1, …, 1000{

1. Randomly draw (N_ind_) participants according to one of two sampling strategies:
  - Uniform sampling: Sample with replacement N_ind_ rows from the airport/runway sleep study simulation data file (n_RWY_∗50 weeks∗10 people) to determine the airport, runway, and number of ANEs (n_event_) for each of the N_ind_ participants
  - Population density-based sampling: Sample N_ind_ runways with each unique runway’s probability of being selected based on its population household unit density. For each of the N_ind_ selected runway, sample from the corresponding airport/runway’s sleep study simulation data file to determine the number of ANEs. Note that population sampling is only available for medium and high traffic runways.
2. For each airport in the sample, draw coefficients from the model distribution for the airport-specific coefficients (B_0_,B_1_)
  c. Mean and variance of this distribution determined by the analysis of the pilot data, as described in step 1 of modeling steps above.
3. For each airport in the sample, randomly select which pilot airport the noise profiles will come from.
  d. For each person in this airport, sample with replacement a given profile of noise events by randomly sampling a person from the chosen pilot airport data; then sample their n_event_i_ noise levels from this profile.
4. *For each person in the sample, generate random effects for b0 and b1.*
  e. Random effects have a mean of 0 and variance is determined by the selected varRE type: Type 1 (empirical average of all pilot data), Type 2 (theoretically inflated by 20% variance), Type 3 (theoretically inflated by 50% variance), as described in step 2 of modeling steps above.
5. Generate the logistic awakening outcome (1 for awakening, 0 otherwise) by flipping a coin with probability determined by Model 2.
6. Reduce simulated data by randomly selecting 30%, then randomly delete 5% of N_ind_ subjects to account for potential missing data.
7. Fit Model 1 to the remaining dataset to obtain estimated coefficients.
8. Save coefficients and other relevant output (e.g., number of ANEs, which airports and runways were selected)
  }
